# Upregulation of the c-MYC oncogene and adjacent long noncoding RNAs PVT1 and CCAT1 in esophageal squamous cell carcinoma

**DOI:** 10.1186/s12885-022-10464-z

**Published:** 2023-01-09

**Authors:** Zahra Roohinejad, Shabbou Bahramian, Fatemeh Tash Shamsabadi, Reza Sahebi, Abolfazl Amini, Davood Sabour, Mohammad Shafiee

**Affiliations:** 1 Genetic Department, University of Medical Sciences, Ganjafrooz Street, Babol, Mazandaran, Iran; 2 Golestan Research Center of Gastroenterology and Hepatology, Golestan University of Medical Sciences, Gorgan, Iran; 3 Department of Medical Biotechnology, School of Advanced Technologies in Medicine, Golestan University of Medical Sciences, Gorgan, Iran; 4 Department of Modern Sciences and Technologies, Faculty of Medicine, Mashhad University of Medical Sciences, Mashhad, Iran

**Keywords:** c-MYC, lncRNAs, PVT1, CCAT1, Esophageal squamous cell carcinoma

## Abstract

**Background:**

All cell types express long non-coding RNAs (lncRNAs), which have the potential to play a role in carcinogenesis by altering the levels of their expression. Squamous cell carcinoma of the esophagus (ESCC) is a deadly disease with a poor prognosis and a high frequency of lymphatic metastases. Understanding the functional role and signaling pathways of two neighboring lncRNAs, CCAT1 and PVT1, in this oncogene’s pathogenesis may help us determine ESCC. Furthermore, it is still unclear whether these lncRNAs are linked to the clinicopathological characteristics of patients with ESCC.

**Methods:**

For this study, we used biopsy from the Imam Khomeini Cancer Institute’s tumor bank in Tehran, Iran to obtain 40 ESCC tumor samples and their normal margin counterparts. The expression levels of the CCAT1, PVT1, and c-MYC genes were assessed using quantitative Real-Time RT-PCR. Additionally, demographic data and clinical-pathologic characteristics, such as tumor grade, tumor stage, lymph node, and metastasis, were taken into consideration. Graphpad prism version 8 was used for bioinformatics analyses.

**Results:**

Comparing ESCC tissues to non-tumor tissues, we found significant upregulation of PVT1, CCAT1, and c-MYC. Patients with ESCC who had increased PVT1 expression also had higher rates of advanced stage and lymph node metastasis, whereas increased CCAT1 expression was only linked to advanced stage and wasn’t associated with lymph node metastasis. In predicting ESCC, CCAT1 (*p* < 0.05) was found to be an important factor. Overall survival was reduced by c-MYC and PVT1 overexpression (*p* < 0.001), according to Kaplan-Meier analysis. PVT1, CCAT1, and c-MYC were found to interact with 23 miRNAs with high and medium score classes, as shown in a bioinformatics study. We summarized the experimentally proven interactions between c-MYC, PVT1, and CCAT1 and other miRNAs, lncRNAs, and proteins.

**Conclusion:**

This is the first report that CCAT1, PVT1 and c-MYC have been found to be up-regulated simultaneously in ESCC. It is possible that these genes may be involved in ESCC as a result of these findings. Therefore, as consequence, more research is needed to determine whether or not these lncRNAs play an oncogenic role in ESCC development and progression, as well as the regulatory mechanisms that control them.

**Supplementary Information:**

The online version contains supplementary material available at 10.1186/s12885-022-10464-z.

## Introduction

Esophageal cancer (EC) is quickly becoming a global public health issue due to a rising mortality rate in the United States and other countries. According to research EC is the sixth leading cause of death due to cancer and the eighth most common form of cancer in the world [1, 2]. More than 90% of EC cases are related with the ESCC sub-type, which includes esophageal squamous cell carcinoma (ESCC) and esophageal adenocarcinoma (EAD). Countries in the European Central Belt, which includes parts of northern China, central Asia, and northern Iran, are the most commonly affected by ESCC [3, 4]. Since current treatments for ESCC are not effective enough to reduce mortality rates, new, effective strategies for rapid diagnosis and targeted treatment have received considerable attention.

Researchers have recently shown a growing interest in noncoding RNAs, which are classified according to length differences: transcripts shorter than 200 nucleotides are known as short/small RNAs (e.g., miRNAs), and those longer than 200 nucleotides are known as long non-coding RNAs (lncRNA) [5–7]. They play critical regulatory roles in a variety of physiological processes, including embryogenesis, stem cell self-renewal, cell cycle, growth, and differentiation [8]. In addition to their role in tumorigenesis and cancer progression, they have also been shown to influence proliferation, metastasis, apoptosis, and drug resistance in previous studies [9]. Oncogenic or tumor-suppressive lncRNAs have recently been discovered to have a huge potential for controlling key tumorigenic pathways by regulating vital genes in normal physiological and/or pathological processes [10, 11].

Because of the presence of the c-MYC gene, the human 8q24 chromosomal locus is a critical genomic region for oncogenesis [12]. c-MYC regulates cell proliferation, differentiation, apoptosis, and angiogenesis, among other processes [13]. The frequent amplification and chromosomal translocation of this locus has been highlighted by recent potentially curative biology. Regulatory elements were also discovered to have a significant impact on c-MYC expression [14]. It appears that the lncRNAs found in this region regulate c-MYC expression at the transcriptional level. Indeed, the induction of these lncRNAs has been linked to the development of cancer [15]. This could make them useful biomarkers for the early detection of cancer. For example, the plasmacytoma variant translocation 1 gene (PVT1) and colon cancer-associated transcript-1 (CCAT1) are located on opposite sides of c-human MYC’s chromosome 8 promoters, respectively. The nine exons in the human PVT1 gene produce a variety of noncoding transcripts of varying lengths [16]. Experimental studies have shown a high expression level of *PVT1* in various cancer types such as breast [16], colon [17], gastric [18], and ovarian cancer [19]. *PVT1* was found in these studies to increase proliferation and metastasis of the cancer cells, suggesting its potential oncogenic role.

The 2795-nucleotide human CCAT1 consists of two exons. CCAT1 knockdown significantly rescued c-MYC expression, while c-MYC up-regulation increased CCAT1 expression, according to recent evidence. Tumorigenesis and metastasis are aided by CCAT1 through a variety of mechanisms in various types of cancer [20, 21].

**Fig. 1:**
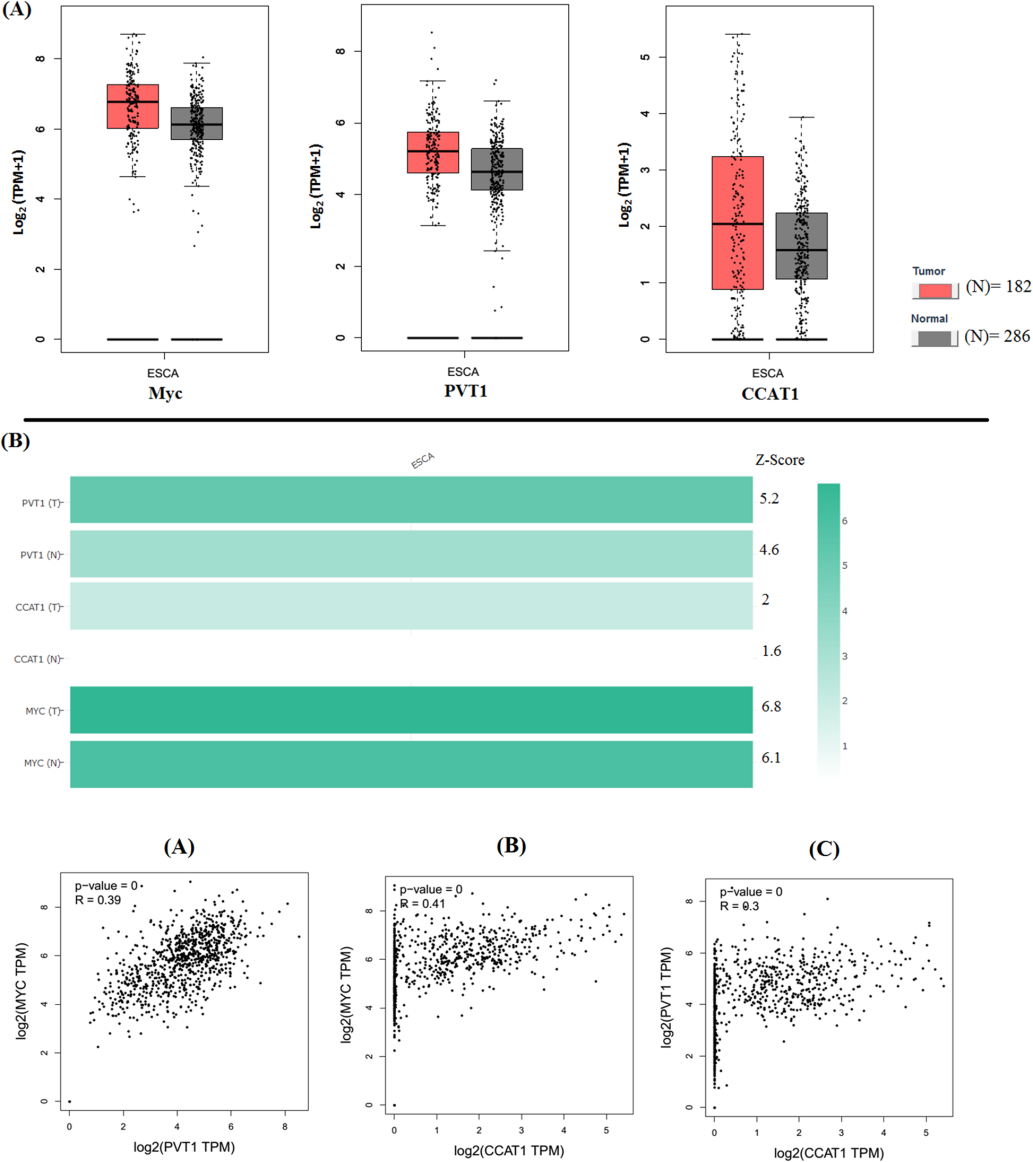
Comparison of expression profile of MYC gene and two neighboring lncRNAs, PVT1 and CCAT1 across esophageal carcinoma samples and adjacent normal tissues. **A** The expression of MYC, PVT1, and CCAT1 are increased in ESCA tissues in comparison to normal tissues, respectively. **B** In comparison to MYC and CCAT1, PVT1 shows a remarkable change in ESCA tissues than normal tissues. **C** Pearson correlation of MYC and PVT1, MYC and CCAT1, as well as CCAT1 and PVT1 genes retrieved from the Gene Expression Profiling Interactive Analysis (GEPIA) resource. Log_2_ (TPM + 1) was used for log-scale. TPM: Transcripts Per Million; ESCA: esophageal carcinoma

The c-Myc gene and two nearby lncRNAs, PVT1 and CCAT1, were found to be highly expressed in esophageal cancer (EC) specimens when compared to nearby normal tissue based on data from The Cancer Genome Atlas (TCGA) extracted from GEPIA. Expression of c-Myc, PVT1 and CCAT1 in EC tissues was higher than in the paired normal tissue. In contrast to the upregulation of CCAT1, the increase in c-MYC protein levels was significantly correlated with the upregulation of PVT1 in EC. Bioinformatics databases were used to better understand the molecular mechanisms controlling c-MYC, PVT1, and CCAT1 (Fig. 1).

## Materials and methods

### Sample collection

40 patients from the Imam Khomeini Hospital in Tehran, Iran, who underwent surgery without local or systemic treatment between 2016 and 2017 had their ESCC tumor tissues and adjacent normal tissues collected. A written consent for sample collection was obtained from patients. However, two samples were lost due to technical difficulties in the process of preparing the data. Frozen tissue specimens were stored at -80 °C for further research. Patients were on average 48 years old (range: 29 to 81). Iran’s Gorgan University of Medical Sciences Research Ethics Committee approved this study (Ethics code: IR.Goums.REC.1395.256).

### Isolation of total RNA

The Trizol reagent (Ambion) was used to extract total RNA from the frozen specimens, as directed by the manufacturer. A260/A280 ratios of between 1.8 and 2.1 were considered for further analysis after RNA quality and quantity were assessed using Nano drop. If a sample is more than 2.1, it will be excluded from the study. RNA was subjected to a DNase enzyme treatment in the absence of RNase to remove any contaminating DNA.

### q-RT-PCR

The quantitative real-time RT-PCR was carried out using standard SYBER green premix EX tag2 (TAKARA) in a 20 µl reaction to detect the mRNA level of *c-MYC*, *PVT1*, and *CCAT1* genes. All qRT–PCR reactions were performed for 40 cycles of 94 °C for 30 s, 60 °C for 30 s, and 72 °C for 30 s, on ABI 7300 Real Time PCR system (Applied Biosystems, USA). Most experiments were performed in triplicate and the specificity of PCR product was confirmed by melt curve analysis. *GAPDH* was chosen as an endogenous control to normalize the expression level of candidate genes. Subsequently, the expression level of target genes was calculated using 2^–△△Ct^ method [22]. The characteristics of primers are indicated in Table 1.

**Table 1:** The primers specifications applied for the expression analysis in q-RT-PCR

Target	Sequences	Amplicon length (bp)
**Myc** (NM_002467.5)	Forward: 5´-CACAGCAAACCTCCTCACAG-3´ Reverse: 5´-GGTGCATTTTCGGTTGTTGC-3´	187
**CCAT1** (NR_108049.1)	Forward: 5´-GGCACTACTCTGTCCCAACA-3´ Reverse: 5´-AGCCATACAGAGCCAACCTG-3´	187
**PVT1** (NR_003367.3)	Forward: 5´-TGAGAACTGTCCTTACGTGACC-3´ Reverse: 5´-AGAGCACCAAGACTGGCTCT-3´	191
**GAPDH** (NM_002046.7)	Forward: 5´-GGTGGTCTCCTCTGACTTCAACA-3´ Reverse: 5´-GTTGCTGTAGCCAAATTCGTTGT-3´	127

### lncRNA-miRNA interaction analysis

The mirDIP 4.1 database (ophid.utoronto.ca/mirDIP/) was used to identify the possible interactions of lncRNAs CCAT1 and PVT1 with miRNAs [23]. This database predicts which MiRNAs have very high, high, and medium score numbers that can target c-MYC, PVT1, and CCAT1. Stronger binding ability was indicated by scores that were both very high.

### Interaction analysis of *c-MYC*, *PVT1* and *CCAT1*

We utilize the EVlncRNA database (biophy.dzu.edu.cn/EVLncRNAs/) in order to identify which genes PVT1 and CCAT1 should target in different cancer types. Furthermore, the RAID v.2 database was used to identify PVT1 and CCAT1 protein interactions. Additionally, the lncRNA Disease database was used to identify CCAT1 and PVT1 mRNA target genes.

### Expression analysis of *c-MYC*, *PVT1* and *CCAT1* in cancer

The lncRNA Disease and Lnc2Cancer (bio-bigdata.net/lnc2cancer) databases were used to investigate the aberrant expression of PVT1 and CCAT1 in various cancers. The potential functional significance of these lncRNAs in other types of cancer was also predicted using target prediction tools like LRLSLDA-LNCSIM1/LRLSLDA-LNCSIM2 [24].

### Statistical analysis

SPSS for Windows version 18 was used for the statistical analysis. Statistical significance was defined as a *p*-value of 0.05 or less. Two-tailed, unpaired samples were used to assess the relationship between lncRNA, PVT1, CCAT1 and c-MYC gene expression and clinical features. Targeted genes were evaluated by plotting receiver operating characteristic (ROC) curves using GraphPad Prism (version 5.0.0.288). The best cutoff value for each group levels to achieve 95% specificity. Also, to divide all samples into two groups (High and low expression), we selected the median expression of PVT1, CCAT1, and MYC as a cutoff point. Analyses were made using Kaplan-Meier and log rank tests to determine the overall survival of the patients.

## Results

### The oncogene *c-MYC* is up-regulated in tumor tissues from ESCC patients

**Fig. 2:**
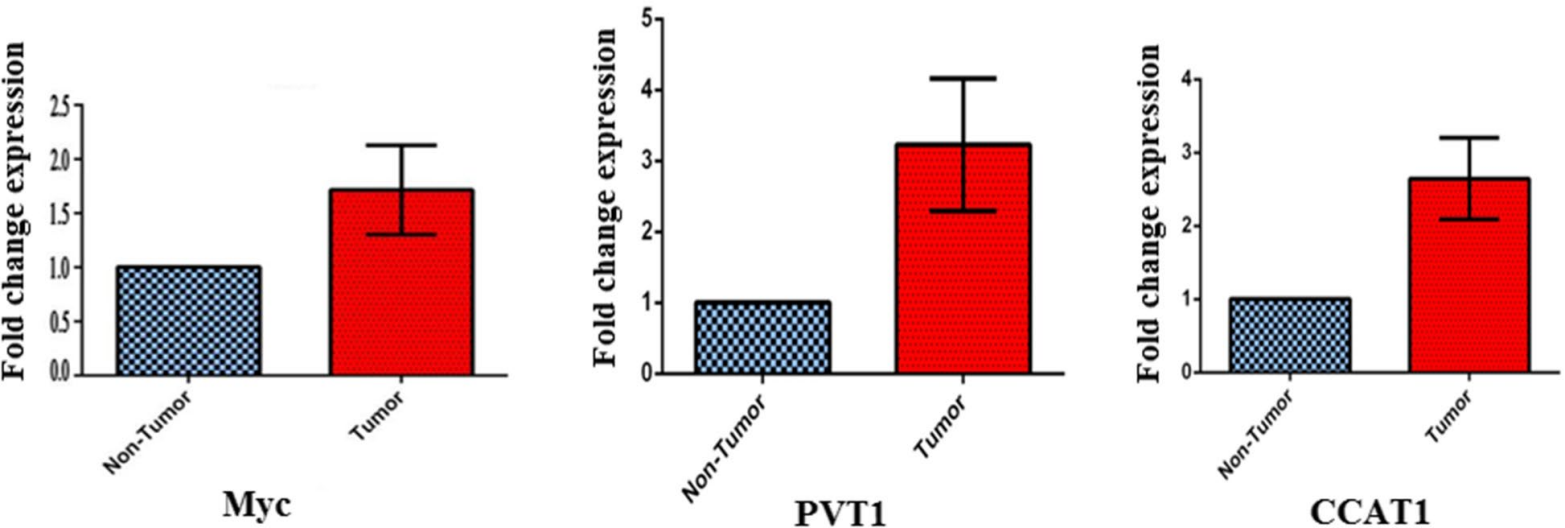
The gene expression profile of MYC gene and two vicinity lncRNAs, PVT1 and CCAT1 across esophagus tumor and paired normal tissues. The transcript level of MYC and two vicinity lncRNAs were significantly increased in esophagus tumor tissues (*p* < 0.05)

We examined the expression of c-MYC in 40 ESCC tumor samples and normal tissues that matched. C-MYC oncogene expression was significantly higher in clinical ESCC specimens than in adjacent non-tumor specimens (*p* = 0.0423; Fig. 2).

### Up-regulation of the lncRNAs *PVT1* and *CCAT1* in ESCC patients

After that, we investigated whether the lncRNAs PVT1 and CCAT1 were expressed differently in tumor tissues taken from ESCC patients to see if there was a link between the two. QRT-PCR results showed that the expression of PVT1 (*p* = 0.0218) and CCAT1 (*p* = 0.0057) transcripts was significantly elevated among ESCC specimens when contrasted with adjacent normal tissues (Fig. 2). In addition, we discovered that the expression of these lncRNAs was linked to the clinical and pathological characteristics of ESCC patients. Patients with advanced ESCC who had high levels of PVT1 or CCAT1 expression were considered to be at a more advanced stage of the disease (Table 1). PVT1 was also linked to increased tumor metastasis, suggesting that this lncRNA plays a pro-metastasis role. LncRNAs may play an important role in the development and progression of ECC, according to these findings (Table 2).

**Table 2:** Association of PVT1 expression with clinicopathological factors in EC patients

Clinical specify	Samples	PVT1 expression		***P*** value
		High	Low	
Age				.468
60<	22	12	10	
60≥	16	7	9	
Gender				.273
Male	20	11	9	
Female	18	8	10	
Tumor size(cm)				.624
5 <	21	13	8	
5 ≥	17	6	11	
Tumor stage				.009
I-II	11	4	7	
III-IV	27	15	12	
Tumor Grade				.064
I-II	8	4	4	
III-IV	30	15	15	
Metastasis				.025
Unknown	9	3	6	
Yes	19	12	7	
No	10	6	4	
Lymph node				.33
Yes	16	7	9	
No	22	12	10	

### *CCAT1* might be a reliable marker for ESCC diagnosis

**Fig. 3:**
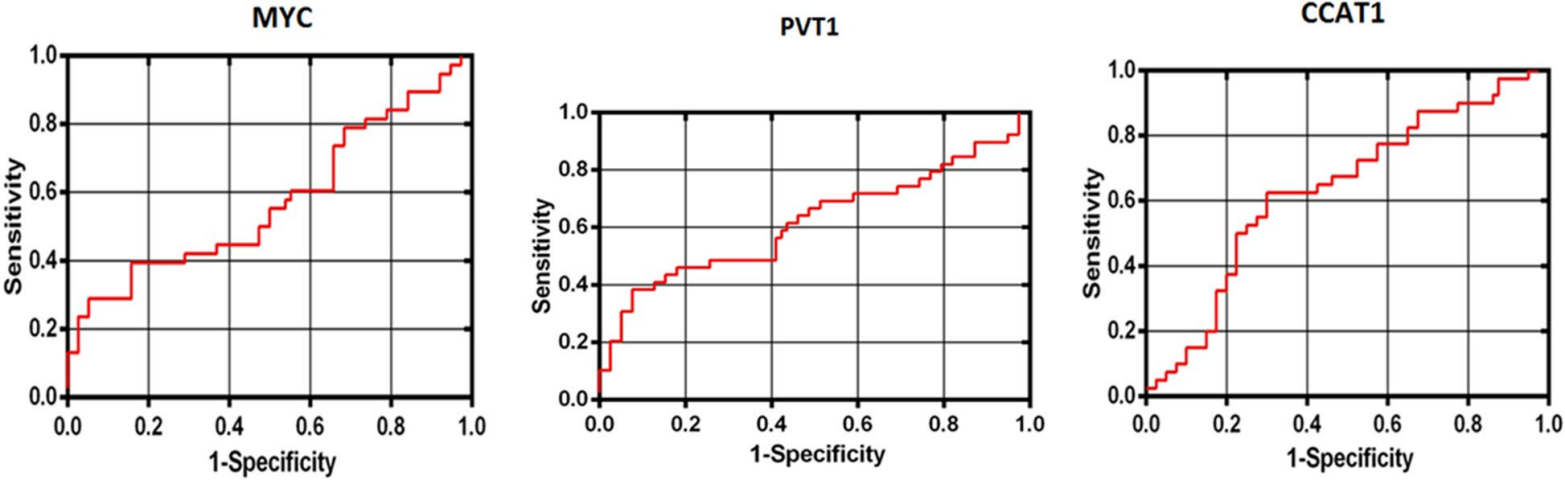
ROC curve analysis to explore the discriminant ability of MYC gene and two vicinity lncRNAs, PVT1 and CCAT1 in predicting esophagus tumor. CCAT1 demonstrated a high degree of suitability for the classification of tumor and non-tumor samples of esophageal tissue samples

The lncRNAs PVT1 and CCAT1 were subjected to ROC curve analysis in order to determine their diagnostic performance. PVT1 and CCAT1 were found to have an area under the receiver operating characteristic curve (AUC) of 0.61 and 0.63, respectively (Fig. 3). As shown in Fig. 3, the value of AUC for *c-MYC* gene was 0.57. This discovery revealed that CCAT1 was a statistically significant discriminating factor in predicting ESCC with a 95% confidence interval for the prediction of the disease (0.51 to 0.75). Indeed, CCAT1 demonstrated a high degree of suitability for the classification of tumor and non-tumor samples of esophageal tissue samples. As a result, the long noncoding RNA (lncRNA) CCAT1 may be a novel biomarker for ESCC detection (Table 3).

**Table 3:** Association of CCAT1 expression with clinicopathological factors in EC patients

Clinical specify	Samples	CCAT1 expression		***P*** value
		High	Low	
**Age**				
60<	22	12	10	.823
60≥	16	11	5	
**Gender**				
Male	20	13	7	.268
Female	18	10	8	
**Tumor size (cm)**				
5 <	21	12	9	.771
5 ≥	17	11	6	
**Tumor stage**				
I-II	11	7	4	.038
III-IV	27	16	11	
**Tumor Grade**				
I-II	8	5	3	.638
III-IV	30	18	12	
**Metastasis**				
Unknown	9	5	4	.345
Yes	19	11	8	
No	10	7	3	
**Lymph node**				
Yes	16	7	9	.409
No	22	16	6	

### Enhanced expression of *c-MYC* and *PVT1* is associated with a decreased overall survival in ESCC patients

**Fig. 4:**
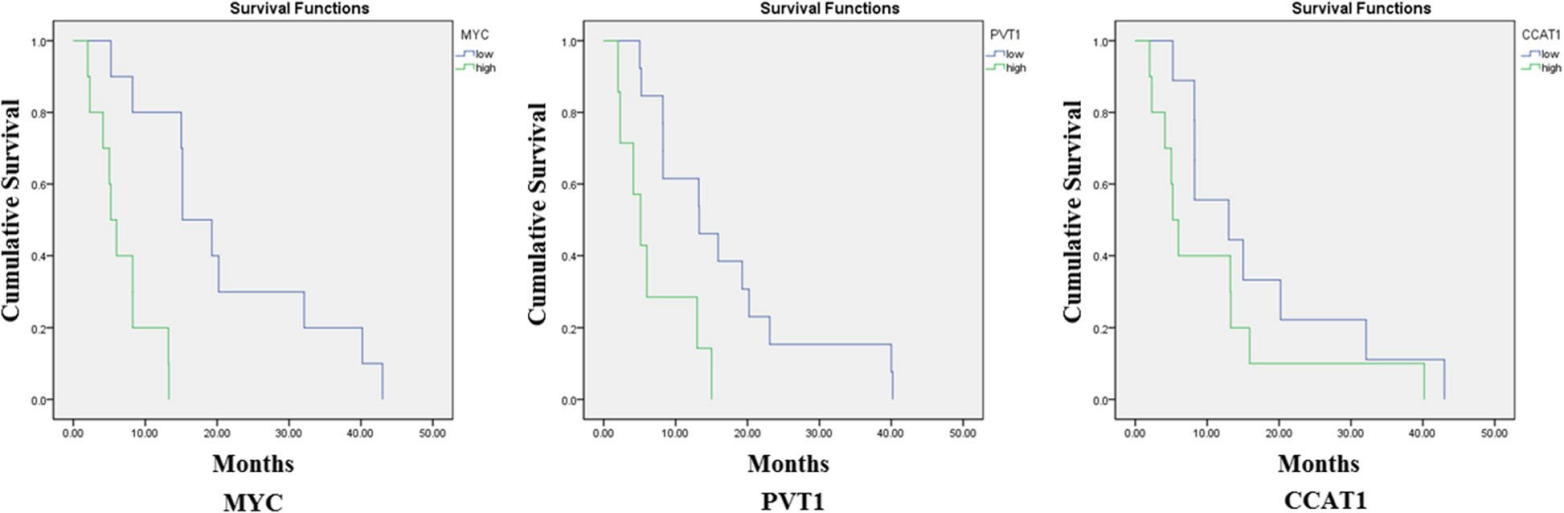
Association of MYC, PVT1, and CCAT1 with survival rate in ESCC patients. Overall survival of patients with high vs. low expression levels of MYC, PVT1, and CCAT1 are illustrated. Patients with high MYC (Chi-Square: 13.46; *p* = 0.000) and PVT1 (Chi-Square: 7.38; *p* = 0.007) expression have poorer survival

Kaplan-Meier analysis was performed to demonstrate the correlation of *c-MYC* gene and lncRNAs *PVT1* and *CCAT1* with survival rate in ESCC patients. The overall survival of patients with the high level of *PVT1* (*p* = 0.007) and *c-MYC* (*p* = 0.00) expression was markedly reduced compared to those with low expression level of these genes (Fig. 4). No significant association was observed between high and low expression of *CCAT1* with survival rate of patients harboring ESCC (*p* > 0.05).

### Human c-MYC interaction with PVT1 and CCAT1

**Fig. 5:**
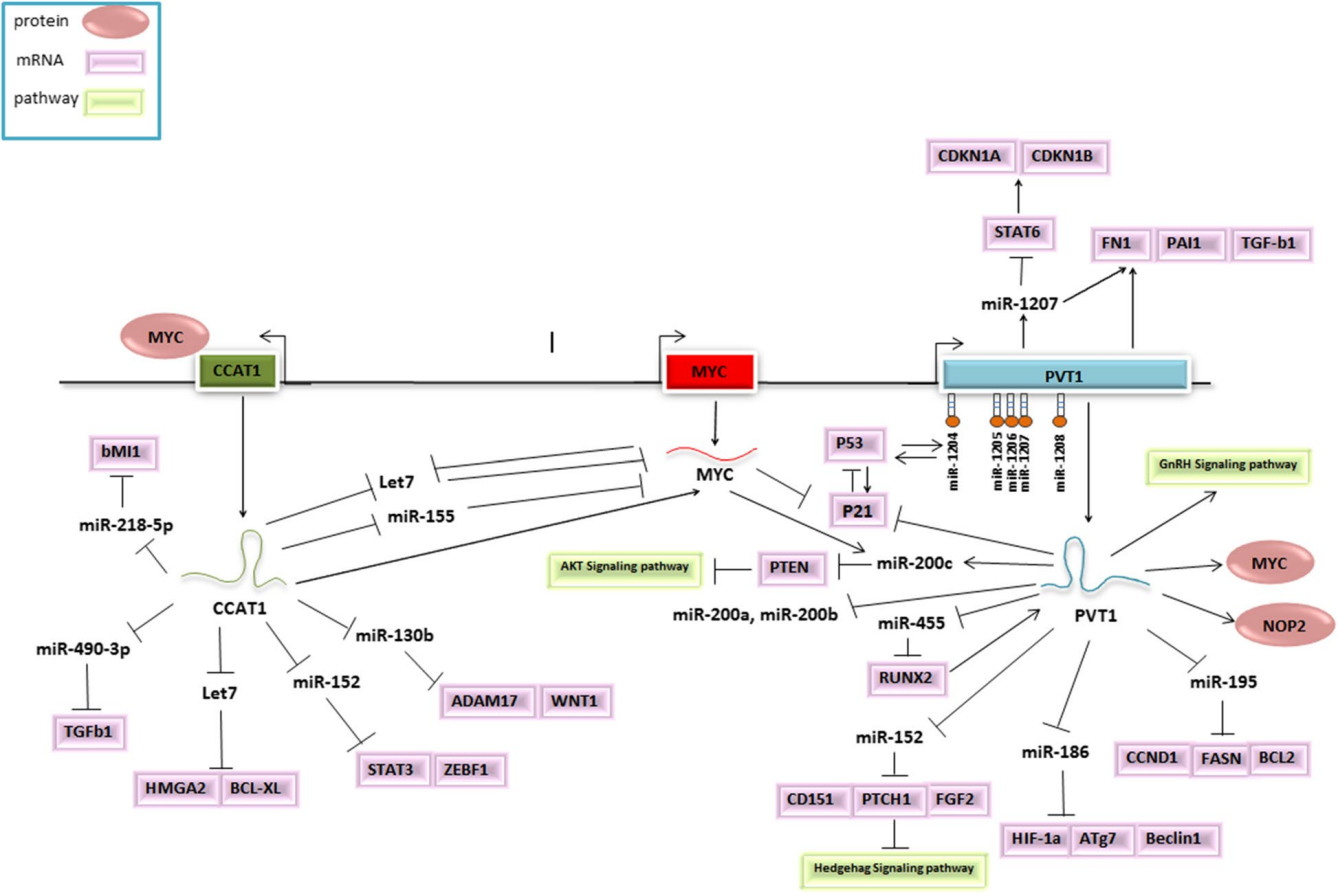
Prognostic significance of human 8q24 chromosomal locus and target gene network in cancers. An illustration of the molecular mechanisms of MYC and its neighboring lncRNAs is shown. lncRNA-miRNA-mRNA interactions in various cancer types have been discovered. There are green and purple rectangles that represent a pathway, respectively. Proteins are depicted as circles. Indicators such as arrows and arrested-line lines show the potential connections among lncRNAs, miRNAs, and mRNA. This is a type of RNA that is both long non-coding and small

The mirIDP 4.1 databases predicted 158 and 139 miRNAs as potential targets of PVT1 and CCAT1, respectively. These miRNAs may all interact with the c-MYC oncogene. 23 miRNAs with high and medium scores may interact with PVT1, CCAT1, and c-MYC. While 24 of these miRNAs interact with PVT1 and c-MYC, 11 of them may be CCAT1 and c-MYC targets (Supplementary Table 1). PVT1 and CCAT1 have been shown to interact experimentally in the EVlncRNA database (Supplementary Table 2). lncRNAs appear to play an important role in a variety of cancers, according to these findings. PVT1 and CCAT1 lncRNA-protein interactions were also extracted from the RAID v.2 resource using the “weak evidence” method (Supplementary Table 3). To be sure, more research is needed to confirm these findings. PVT1, CCAT1, and MYC have been shown to interact with microRNAs, lncRNAs, and proteins, which includes a summary of all the evidence for these interactions (Fig. 5).

### The lncRNAs *PVT1* and *CCAT1* are up-regulated in various types of cancer

Following that, we investigated whether the lncRNAs PVT1 and CCAT1 are similarly overexpressed in other tumor types. PVT1 and CCAT1 were implicated in the pathogenesis of certain cancers, according to data extracted from the Lnc2Cancer and LncRNA Disease databases. Numerous molecular experiments have been performed to validate this information, including FISH, RIP, RT-PCR, Western blot, Dual luciferase reporter gene assay, qRT-PCR, RNAi, and MTT. Additionally, the LRLSLDA-LNCSIM1 and LRLSLDA-LNCSIM2 methods have been used to predict the role of both lncRNAs in certain cancers. Table 4 lists cancers that experimentally interfere with both lncRNAs, PVT1 and CCAT1. This table categorizes evidence as experimental or expected. Indeed, these two long noncoding RNAs may contribute to the pathogenesis of predicted cancers. Different cancers were compared for lncRNA expression patterns across tumor and non-tumor samples (Supplementary Fig. 1). Cancers that had increased levels of PVT1 lncRNA included ESCA, DLBC, COAD, CHOL, BRCA, READ, SARC, LUSC, LUAD, KIRC, GBM, and STAD. There was a marked decrease in the level of PVT1 in the tumor tissues of LAMC, OV, ACC, THCA, and TGCT compared to the non-tumor tissues. Even in cancerous samples such as READ, COAD, ESCA, LUSC, STAD, and CHOL, CCAT1 was found to be highly up-regulated. Only two types of cancer, HNSC and LIHC, showed lower levels of CCAT1 expression than normal. The anatomical position, stage, and histological characteristics of the tumor all affect how risk is classified for HNSCC and LIHCC.

**Table 4:** Experimental validated and predicted cancers for PVT1 and CCAT1 interference

lncRNA	Detection Method	Cancers Name
**CCAT1**	**Experimental**	esophageal squamous cell carcinoma, breast cancer, colon cancer, gallbladder cancer, Glioma, hepatocellular carcinoma, lung cancer, ovarian cancer, pancreatic cancer, retinoblastoma, Nasopharyngeal carcinoma, stomach cancer, colorectal cancer, cervical cancer, osteosarcoma, renal cell carcinoma, colorectal cancer
	**Predicted**	Lymphoma, thyroid cancer, urinary bladder cancer
**PVT1**	**Experimental**	esophageal squamous cell carcinoma, thyroid cancer, lung cancer, renal carcinoma, lung squamous cell carcinoma, bladder urothelial carcinoma, B-cell lymphoma, lymph node metastasis, plasmacytoma, osteosarcoma, multiple myeloma, colon cancer, nasopharyngeal cancer, cervical cancer, pancreatic cancer, colorectal cancer, breast cancer, gastric cancer, non-small cell lung cancer
	**Predicted**	endometrial cancer, germ cell cancer, leukemia, liver cancer, lung cancer, testicular cancer, tongue cancer, acute lymphocytic leukemia, adrenocortical carcinoma, basal cell carcinoma, glioblastoma, lymphoblastic leukemia, parotid gland cancer, rhabdomyosarcoma

Data obtained from LncRNADisease v2.0 database

## Discussion

One of the most serious public health issues is EC. Survival after five years is still quite low because of this. As a result, new biomarkers for early detection and targeted treatment of EC must be discovered. LncRNAs have been shown to play a critical role in the development of cancer and other pathological processes, as evidenced by previous research [5]. For instance, *PVT1* has been shown to promote cervical cancer progression by silencing miR-200b [25] and support colorectal cancer cells by inhibiting apoptosis [17]. CCAT1 promotes the progression of hepatocellular carcinoma and gallbladder cancer by acting as a let-7 or miRNA-218 sponge, respectively [26, 27]. It also promotes proliferation and migration of pancreatic cancer cells by upregulating c-*MYC* [21]. The same function was observed in esophageal carcinoma by regulating SPRY4 and HOXB13 expression [28].

Thus, the attention of research is shifting to the roles of dysregulated lncRNA expression in various cancers [11, 29]. Cancer detection could benefit greatly from a better understanding of the co-expression network of lncRNAs and protein-coding genes. Cancer and other diseases can be caused by translocations and amplification of the 8q24 locus because it is susceptible to these events [30, 31]. In addition, a number of critical regulatory elements can be found in this region, the absence of which can lead to the development of cancer. It is common for MYC, a cancer-promoting oncogene, to be activated and expressed abnormally in various cancers [32, 33].

This research set out with the aim of assessing the expression of *MYC* gene along with two vicinity lncRNAs, *PVT1* and *CCAT1*, in ESCC patients. *PVT1* is an oncogenic lncRNA which has a critical role in cancer development and pathophysiology. Our results revealed that the expression levels of *PVT1* in ESCC samples were significantly higher than paired adjacent normal tissues. Numerous studies have reported that the over-expression of *PVT1* is implicated in the development of different cancer types such as gastric cancer [18], colorectal cancer [17], non-small cell lung cancer [34], ovarian and breast cancer [35]. *PVT1* was also reported to be over-expressed in hepatocellular carcinoma [36], playing a key role in cancer cell proliferation, metastasis, and cell cycling. Notably, *PVT1* knockdown led to a remarkable loss of lung and colorectal cancer cell proliferation and invasion in vitro [17, 34].

CCAT1 was also found to be up-regulated in ESCC tissues compared to normal tissues on the margin. This finding backs up previous research that looked at colorectal cancer tissues and peripheral blood samples from the same patients, but not healthy people [37]. This lncRNA was found to be highly expressed in stomach adenocarcinoma [18]. CCAT1 expression was found to be significantly higher in breast cancer tissues in another study [38]. In gastric cancer tissues, CCAT1 levels were significantly higher than those in adjacent normal tissues [39]. CCAT1 was shown to increase the proliferation and migration of tumor cells when over-expressed, but when knocked down, these properties were reduced. The amplification and translocation of the 8q24 region may be responsible for the upregulation of PVT1 in a variety of cancers. The PVT1 locus contains a cluster of miRNAs, including miR-1204, miR-1205, miR-1206, and miR-1208. Increased levels of PVT1 transcript are a result of PVT1 controlling MYC protein stability. Proliferation of cancer cells can be boosted by the simultaneous expression of MYC and PVT1. In HER2-positive breast cancer patients, the co-expression of *c-MYC* and PVT1 has been shown to play an important role in promoting tumor malignancy [40]. However, in breast and ovarian cancers, PVT1 acts independently of MYC, acting as an anti-apoptotic protein [41]. In order to induce apoptosis, *c-MYC* alone had to be inhibited [35]. However, it has been proposed that up-regulation of PVT1 locus-embedded miRNAs independent of *c-MYC* has anti-proliferation and apoptotic functions [41, 42]. Therefore, these miRNAs may play a role in PVT1’s oncogenic properties. Another study found that CCAT1 silencing reduced *c-MYC* transcription and chromatin loops interaction in this locus. This is because the E-box element of the CCAT1 promoter can be directly bound by *c-MYC*, resulting in both transcriptional activation and expression of CCAT1 [39]. Because CCAT1 expression is linked to *c-MYC* upregulation, and *c-MYC* deregulation has a significant impact on cancer proliferation and migration, it can be classified as a tumor oncogene. Furthermore, we also found that the up-regulation of *PVT1* was associated with advanced clinical stage and distant metastasis. These results are consistent with those observed in earlier studies which indicated that. A previous study has reported that the increased expression of *PVT1* was associated with enhanced invasion of gastric cancer cells to lymph nodes [18]; they suggested *PVT1* as a new biomarker and therapeutic target for gastric cancer. In accordance with our findings, the up-regulation of *PVT1* in lung cancer was found to be positively correlated with clinical tumor stage, lymph nodes and distance metastasis [34].

In this study, the high expression level of *CCAT1* was positively correlated with clinical tumor stage. So far, several findings have revealed that the up-regulation of *CCAT1* is associated with different clinical parameters such as tumor grade, lymph node metastasis, and clinical tumor stage. For instance, the increased level of *CCAT1* in gastric cancer tissues was positively correlated with the tumor size and lymph node metastasis [39]. The same observations regarding *CCAT1* expression status was reported in breast cancer which showed a positive correlation with differentiation grade, stage, and lymph nodes metastasis [38].

Furthermore, the molecular mechanism of targeted lncRNAs, PVT1 and CCAT1, in tumors was performed by combining the results of published studies and using online bioinformatics resources. Comprehensive networks of lncRNA-miRNA-mRNA can regulate several important cell functions. In this regulatory network, lncRNAs play a vital role in controlling gene expression by participating in the ceRNA, RNA-RNA and RNA-proteins interactions. Thus, the aberrant expression of this lncRNA-mediated regulatory network has an important role in cancer pathogenesis.

For instance, miR-1204 increases the expression of p53 by sharing promoter and regulatory elements with *PVT1*, thereby potentially promoting apoptosis and cell cycle arrest [43]. miR-1207 leads to breast cancer cell progression by inhibiting STAT6 which acts as an activator of the cell cycle-dependent kinase inhibitors [44].

The interaction of *PVT1* with miR-200 family is another example of what it has been shown that miR-200a and miR-200b are direct targets of *PVT1* in non-small cell lung cancer. Also, increased methylation of miR-200b promoter through binding of *PVT1* with EZH2 inhibits the expression of miR-200b [25]. Abnormal expression of this miRNA increased cell proliferation and migration. Likewise, direct interaction of *PVT1* and *c-MYC* with miR-200c promoter was found to induce the expression of miR-200c, resulting in activation of AKT signaling pathway by inhibition of PTEN [45].

Moreover, *PVT1* induces HIF1α and RUNX2 expression by suppressing miR-186 [46], and miR-455 [47], respectively. It was also reported that *PVT1* activates CD151 and FGF2 expression through suppression of miR-152 in gastric cancer [48]. The up-regulation of *CCAT1* induces cell proliferation, migration, and inhibition of apoptosis by enhancement of MYC and HMGA2 expression while reducing the expression of let-7 miRNAs [26, 49]. Moreover, *CCAT1* has a potential role in inhibiting ADAM17 and WNT1 via targeting miR-152 and promoting STAT3 and ZEB1 expression by targeting miR-130a [50]. This lncRNA also promotes cancer malignancy by inhibiting miR-218 in gallbladder cancer [27].

## Conclusion

lncRNAs have been shown to play critical roles in a variety of physiological and pathological contexts in their interactions with other molecular players. PVT1 and CCAT1 lncRNA and the MYC oncogene expression patterns in ESCC patients have been studied for the first time in this study. Patients with advanced tumor stage and metastasis were more likely to have elevated levels of PVT1 and CCAT1. As a result, these lncRNAs (in particular CCAT1) may make excellent biomarkers for the early detection of ESCC. Our findings suggest that lncRNAs near oncogenes may play an important role in cancer cell development and malignancy regulation. These pro-tumor lncRNAs, therefore, have a significant role in the pathophysiology of ESCC, which necessitates further investigation into the molecular mechanisms that govern their function, which could lead to the development of reliable diagnostic biomarkers or targeted therapies for the disease.

## Supplementary Information

**Additional file 1.**

## Data Availability

The data that support the findings of this study are available from the corresponding author upon reasonable request. The datasets generated and/or analysed during the current study are available in the Golestan University of Medical Sciences, Gorgan, Iran.
