# Supplementary material for: Upregulation of the c-MYC oncogene and adjacent long noncoding RNAs PVT1 and CCAT1 in esophageal squamous cell carcinoma
Source: BMC Cancer. 2023 Jan 9;23:34. doi: 10.1186/s12885-022-10464-z (PMC9830801; doi:10.1186/s12885-022-10464-z)
Supplement: Supplementary file 1 — Additional file 1. [file 12885_2022_10464_MOESM1_ESM.docx]

**Bioinformatics analysis**

To understand the molecular mechanism underlying the control of MYC, PVT1, and CCAT1, some online bioinformatics databases were used. Based on these data, we demonstrated the expression profile of each genes signaling pathway and lncRNA-microRNA-mRNA interaction.

Table 1. The most common interaction of c-MYC, PVT1 and CCAT1 with microRNAs

| **PVT1** | **MYC** | **CCAT1** | **Score** |
| --- | --- | --- | --- |
| hsa-miR-214-3p | hsa-miR-214-3p |  | H M |
| hsa-miR-24-3p | hsa-miR-24-3p |  | M H |
|  | hsa-miR-34b-5p | hsa-miR-34b-5p | VH M |
|  | hsa-let-7c-5p | hsa-let-7c-5p | VH M |
|  | hsa-let-7a-5p | hsa-let-7a-5p | H M |
|  | hsa-miR-296-3p | hsa-miR-296-3p | H M |
| hsa-miR-145-5p | hsa-miR-145-5p |  | M H |
|  | hsa-miR-98-5p | hsa-miR-98-5p | H M |
|  | hsa-let-7f-5p | hsa-let-7f-5p | H M |
| hsa-miR-449c-5p | hsa-miR-449c-5p | hsa-miR-449c-5p | M H M |
| hsa-let-7e-5p | hsa-let-7e-5p | hsa-let-7e-5p | M H M |
| hsa-let-7i-5p | hsa-let-7i-5p | hsa-let-7i-5p | M H M |
|  | hsa-let-7d-5p | hsa-let-7d-5p | H M |
|  | hsa-miR-196a-5p | hsa-miR-196a-5p | H M |
|  | hsa-miR-940 | hsa-miR-940 | H M |
| hsa-miR-374a-5p | hsa-miR-374a-5p |  | M H |
|  | hsa-miR-629-5p | hsa-miR-629-5p | H M |
| hsa-miR-374b-5p | hsa-miR-374b-5p |  | M H |
| hsa-miR-190a-5p | hsa-miR-190a-5p |  | M H |
| hsa-miR-190b | hsa-miR-190b |  | M H |
| hsa-miR-186-5p | hsa-miR-186-5p |  | M H |
|  | hsa-miR-376c-3p | hsa-miR-376c-3p | M M |
| hsa-miR-216a-5p | hsa-miR-216a-5p | hsa-miR-216a-5p | M M M |
| hsa-miR-20b-5p | hsa-miR-20b-5p | hsa-miR-20b-5p | M M M |
| hsa-miR-143-3p | hsa-miR-143-3p | hsa-miR-143-3p | M M M |
| hsa-miR-519d-3p | hsa-miR-519d-3p |  | M M |
| hsa-miR-93-5p | hsa-miR-93-5p |  | M M |
| hsa-miR-152-3p | hsa-miR-152-3p | hsa-miR-152-3p | M M M |
| hsa-miR-106a-5p | hsa-miR-106a-5p |  | M M |
| hsa-miR-140-5p | hsa-miR-140-5p | hsa-miR-140-5p | M M M |
| hsa-miR-455-5p | hsa-miR-455-5p |  | M M |
| hsa-miR-383-5p | hsa-miR-383-5p |  | M M |
| hsa-miR-128-3p | hsa-miR-128-3p |  | M M |
| hsa-miR-186-5p | hsa-miR-186-5p |  | M M |
| hsa-miR-9-5p | hsa-miR-9-5p |  | M M |
| hsa-miR-16-5p | hsa-miR-16-5p |  | M M |
| hsa-miR-195-5p | hsa-miR-195-5p |  | M M |
| hsa-miR-365a-3p | hsa-miR-365a-3p |  | M M |
| hsa-miR-130a-3p | hsa-miR-130a-3p |  | M M |
| hsa-miR-150-5p | hsa-miR-150-5p | hsa-miR-150-5p | M M M |
| hsa-miR-488-3p | hsa-miR-488-3p |  | M M |
| hsa-miR-377-3p | hsa-miR-377-3p |  | M M |
| hsa-miR-590-5p | hsa-miR-590-5p |  | M M |
| hsa-miR-149-5p | hsa-miR-149-5p | hsa-miR-149-5p | M M M |
| hsa-miR-29a-3p | hsa-miR-29a-3p | hsa-miR-29a-3p | M M M |
| hsa-miR-148a-3p | hsa-miR-148a-3p | hsa-miR-148a-3p | M M M |
| hsa-miR-148b-3p | hsa-miR-148b-3p | hsa-miR-148b-3p | M M M |
| hsa-miR-106b-5p | hsa-miR-106b-5p |  | M M |
| hsa-miR-29b-3p | hsa-miR-29b-3p |  | M M |
| hsa-miR-29c-3p | hsa-miR-29c-3p |  | M M |
| hsa-miR-224-5p | hsa-miR-224-5p |  | M M |
| hsa-miR-221-3p | hsa-miR-221-3p | hsa-miR-221-3p | M M M |
| hsa-miR-222-3p | hsa-miR-222-3p | hsa-miR-222-3p | M M M |
| hsa-miR-212-3p | hsa-miR-212-3p | hsa-miR-212-3p | M M M |
| hsa-miR-127-3p | hsa-miR-127-3p | hsa-miR-127-3p | M M M |
| hsa-miR-216b-5p | hsa-miR-216b-5p |  | M M |
| hsa-miR-217 | hsa-miR-217 | hsa-miR-217 | M M M |
| hsa-miR-215-5p | hsa-miR-215-5p | hsa-miR-215-5p | M M M |
|  | hsa-miR-1206 |  | M |
|  | hsa-miR-1207-5p |  | M |
|  | hsa-miR-1208 |  | M |
| hsa-miR-298 | hsa-miR-298 | hsa-miR-298 | M M M |
| hsa-miR-210-3p | hsa-miR-210-3p | hsa-miR-210-3p | M M M |
| hsa-miR-2110 | hsa-miR-2110 | hsa-miR-2110 | M M M |
| hsa-miR-199b-5p | hsa-miR-199b-5p | hsa-miR-199b-5p | M M M |

Note: Interaction score was identified by VH: very high, H: high, and M: moderate. Data retrieved from mirIDP4.1 database.

Table 2. Experimental interactions of PVT1 and CCAT1 in various cancers

| **PIMD** | **Cancer Name** | **Level of**  **interaction** | **Interaction**  **target** | **lncRNA**  **Name** |
| --- | --- | --- | --- | --- |
| 25569100 | gallbladder | RNA-RNA | miRNA-218-5p | **CCAT1** |
| 26825578 | gastric cancer | RNA-RNA | miR-490 | **CCAT1** |
| 2518565 | colon cancer tissues | DNA-TF | c-Myc | **CCAT1** |
| 23143645 | gastric carcinoma | DNA-TF | c-Myc | **CCAT1** |
| 25884472 | hepatocellular carcinoma | RNA-RNA | let-7a-b-c | **CCAT1** |
| 25569100 | gallbladder | RNA-Protein | BMI1 | **CCAT1** |
| 26064266 | colorectal cancer | RNA-Protein | E- & N-cadherin | **CCAT1** |
| 17503467 | neuroblastoma cells | DNA-TF | c-Myc | **PVT1** |
| 17503467 | neuroblastoma cells | RNA-DNA | c-Myc | **PVT1** |
| 22110125 | cancer | DNA-TF | P53 | **PVT1** |
| 25890171 | gastric cancer | RNA-Protein | EZH2 | **PVT1** |
| 26427660 | thyroid cancer | RNA-Protein | Cyclin D1 & TSHR | **PVT1** |
| 26908628 | Non-Small Cell Lung Cancer | RNA-DNA | LATS2 | **PVT1** |
| 21814516 | prostate cancer | RNA-DNA | YY1 | **PVT1** |

Note: The interaction targets of CCAT1 and PVT1, tumor type and the published article number ID are extracted from EVLncRNAs resource. TF: Transcription factor.

Table 3. RNA-Protein interactions of PVT1 and CCAT1

| **RNA-Protein interaction** | **RNA-Protein interaction** | **RNA-Protein interaction** |
| --- | --- | --- |
| PVT1- UPF1  PVT1- POU5F1  PVT1- CEBPB  PVT1- E2F4  PVT1- SP1  PVT1- CEBPB  PVT1- TFAP2A  PVT1- CDX2  PVT1- POU5F1  PVT1- FOXP2  PVT1- MAX  CCAT1- PTBP1  CCAT1- TIAL1  CCAT1- TIA1  CCAT1- U2AF2  CCAT1- HNRNPC  CCAT1- FUS  CCAT1- EIF4A3  CCAT1- UPF1 | PVT1- E2F6  PVT1- POU2F2  PVT1- SMAD3  PVT1- EIF4A3  PVT1- AR  PVT1- TCF7L2  PVT1- PTBP1  PVT1- NRF1  PVT1- U2AF2  PVT1- ZNF263  PVT1- FUS  PVT1- SMAD4  PVT1- SMARCC2  PVT1- TIA1  PVT1- HNRNPC  PVT1- IRF4  PVT1- TAF1  PVT1- STAT1  PVT1- ERG  PVT1- FOXA1 | PVT1- SMARCA4  PVT1- NFE2  PVT1- EBF1  PVT1- JUN  PVT1- PPARG  PVT1- TFAP2A  PVT1- CDX2  PVT1- FOSL2  PVT1- ZZZ3  PVT1- FOS  PVT1- FLI1  PVT1- CEBPA  PVT1- NR3C1  PVT1- EWSR1  PVT1- RAD21  PVT1- NANOG  PVT1- GATA6  PVT1- REST  PVT1- GATA2  PVT1- DGCR8  PVT1- SMARCB1 |

Note: The interaction of PVT1 and CCAT1 lncRNAs with proteins were extracted based on the 'Weak Evidence’ method from RAID v2.0 database.

PVT1


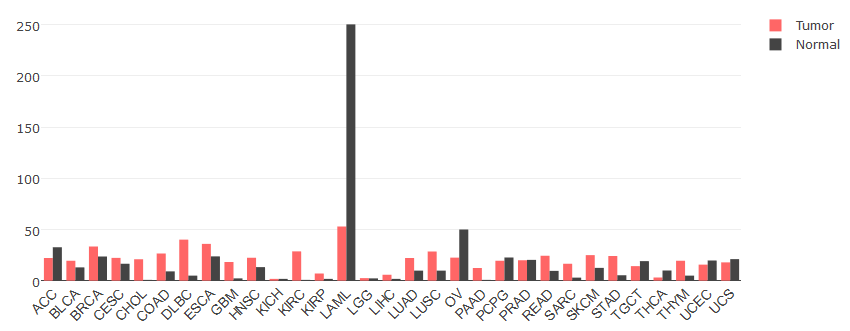


CCAT1


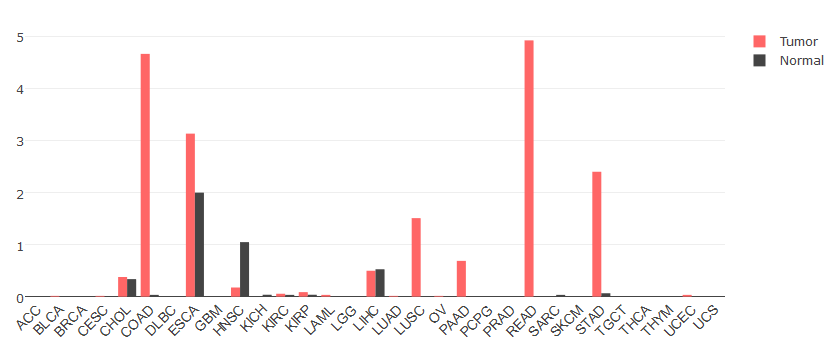


Figure 1. PVT1 and CCAT1 lncRNAs expression profile across tumor and paired normal tissues from GEPIA (bar plot). The height of the bar represents the median expression of certain tumor types or normal tissues. (A) The level of PVT1 expression was compared in different tumor tissues than in adjacent normal tissues; (B) the expression profile of CCAT1 across different tumors and adjacent normal tissues. The up-regulation of both lncRNAs, PVT1 and CCAT1, was observed in READ, COAD, ESCA, LUSC, and CHOL cancer types.
